# Scope of services provided to childhood cancer patients by the Brazilian Pediatric Palliative Care Network

**DOI:** 10.3389/fonc.2024.1376631

**Published:** 2024-06-20

**Authors:** Esther Angélica Luiz Ferreira, Cristina Ortiz Sobrinho Valete, Silvia Maria de Macedo Barbosa, Graziela de Araujo Costa, Poliana Cristina Carmona Molinari, Ana Cristina Pugliese de Castro, Simone Brasil de Oliveira Iglesias, Maycon Rodrigo Sarracini, Rodrigo Bezerra de Menezes Reiff, Cristina Helena Bruno, Maria Sagrario Gómez-Cantarino, Ana María Ullán

**Affiliations:** ^1^ Department of Medicine, Federal University of São Carlos, São Paulo, Brazil; ^2^ Center for Studies in Pain and Palliative Care, Federal University of São Carlos, São Paulo, Brazil; ^3^ Children’s Institute of the Hospital das Clínicas of the Faculty of Medicine of the University of São Paulo, São Paulo, São Paulo, Brazil; ^4^ Sírio-Libanês Hospital, São Paulo, São Paulo, Brazil; ^5^ Faculty of Medicine of Jundiaí, Jundiaí, São Paulo, Brazil; ^6^ Department of Pediatrics, Federal University of São Paulo, São Paulo, São Paulo, Brazil; ^7^ Department of Physical Education and Human Motricity, Federal University of São Carlos, São Carlos, São Paulo, Brazil; ^8^ Central Paulista University Center, São Carlos, São Paulo, Brazil; ^9^ University of Castilla-La Mancha, Toledo, Spain; ^10^ University of Salamanca, Salamanca, Spain

**Keywords:** medical oncology, cancer pain, pediatric palliative care, palliative medicine, pediatrics, medical education, Brazil

## Abstract

**Introduction:**

Brazil is a developing and an Upper Middle Income, categorized by the World Bank. Therefore, it is a country that needs a special vision for children with oncological diseases who require Pediatric Palliative Care. This study aimed to understand the specificities of services that provide oncology services in comparison to those that do not provide oncological care.

**Methods:**

This is a descriptive, cross-sectional, and online survey study. A questionnaire was created by a multidisciplinary group of leaders from the Brazilian Pediatric Palliative Care Network and then the survey was distributed using a snowball strategy.

**Results:**

Of the 90 services that answered the questionnaire, 40 (44.4%) attended oncologic patients. The Southeast represented most of the services (57.57%), followed by the Northeast, with 18.89% (17 services), the South with 12.22% (11 services), and the Center West with 8.89% (8 services). No differences were observed in access to opioid prescriptions between the services. It was observed that those services that attended oncologic patients had a tendency to dedicate more time to Pediatric Palliative Care.

**Discussion:**

The distribution of services that cover oncology and those that do not, are similar in the different regions of Brazil. In Brazil, there are difficulties in accessing opioids in pediatrics: access to opioid prescriptions without differences revealed that even pediatric oncologists might have difficulty with this prescription, and this should improve. It is concluded that education in Pediatric Palliative Care is the key to improvements in the area.

## 1 Introduction

After accidents, cancer is the second-highest cause of death in children aged 1 to 14 in the world (1). In general, from a global point of view, although rare compared with the adult population, cancer remains the leading cause of death by disease in children (2). According to some authors, approximately 15,590 children were diagnosed with cancer in 2018 in the United States (2) and every year, around 400 000 children and adolescents aged 0 to 19 are diagnosed with cancer in the world (1).

Palliative Care is defined as assistance offered to prevent and alleviate the suffering of adult and pediatric patients and their families who face problems associated with potentially fatal illnesses, including the physical, psychological, social and spiritual suffering of patients and their families (3). Children with cancer experience physical symptoms and a reduced quality of life (1). A child’s suffering affects the entire family, as well as the society that surrounds them (3). In this scenario, Pediatric Palliative Care (PPC) emerges as an integral form of assistance for these patients and their families (4). PPC implies early identification of the degree of serious disease involved, evaluation, and adequate treatment, improving quality of life, promoting dignity and comfort, without accelerating or delaying death, and may even positively influence the course of the disease, an essential aspect for the prognosis of the pediatric patient (5).

Cancer constitutes 5.2% of the palliative care needs of children in the world (1). Approximately, 90% of children with cancer live in low- and middle-income countries, constituting 84% of the global burden of childhood cancers (1). Children in low- and middle-income countries have low cure rates and high death rates, making palliative care relevant in a pediatric oncology setting (1). Brazil is a developing and Upper Middle Income country, categorized by the World Bank, but, mainly, it is a country of large territorial extension, with multiple cultures, accompanied by financial disparities (3). Therefore, an individualized vision for the realities of children with oncological diseases who have indications that PPC is necessary, which is a great challenge (4).

Palliative Care services are those health services (such as hospitals and clinics) that provide assistance to children who need this area. In 2019, in Brazil, of the 191 Palliative Care services evaluated, only 40.3% were qualified for the care of children and adolescents (4, 6): although more than 40% of palliative care services in Brazil served children and adolescents, it was not known exactly what type of assistance was provided. As for public policies in Brazil, some states have already published their own normative on the topic (7). It is worth remembering that the specificities in pediatrics are unique and should be considered in order to introduce an appropriate policy (8, 9). Measuring the need and capacity to provide PPC are key elements in care planning for a country or region (10). In 2019, the International Children’s Palliative Care Network (ICPCN) published estimated levels of provision of children’s palliative care worldwide, based on information available in the literature (11). These levels can range from 1 to 5, from lowest to highest, and Brazil was then classified as level 3, defined as “Evidence of localized palliative care provision for children and availability of training” (11): this classification was not carried out directly with Brazilian representatives, as data had never been collected directly in this country.

Therefore, this study aimed to map PPC services in Brazil, studying their formations and particularities, understand the composition of the teams, the services provided and whether they supported children diagnosed with pediatric cancer.

## 2 Materials and methods

### 2.1 Scenario

This is a descriptive, cross-sectional, and online survey study. A quantitative questionnaire in Brazilian Portuguese was created by a multidisciplinary group of leaders from the Brazilian Pediatric Palliative Care Network and then the survey was distributed using a snowball strategy.

The Brazilian Pediatric Palliative Care Network has been working since October 2020. It is a collaborative research project developed by the Medicine Department of São Carlos Federal University, in the interior of São Paulo. It is a formalized network that focuses on the promotion, spread, and dissemination of knowledge in Pediatric Palliative Care and is a multidisciplinary initiative. Many Brazilian services participate and compound the study groups in the network. There were, at that time, 40 services that provide assistance to children, that is, between zero and 18 years old, represented in the network organization. The network leaders are healthcare professionals involved directly in assistance, teaching, and research in PPC in Brazil, and some are the leaders of the PPC group in the Brazilian Society of Pediatrics.

### 2.2 Criteria

A convenience sample is a type of non-probability sampling method in which the sample is drawn from a group of people who are easy to contact or reach. As this was the first study carried out in Brazil, a sample size calculation was not carried out, but instead, convenience sampling was captured, using a snowball strategy. Fifteen online invitations were sent by the Brazilian Pediatric Palliative Care Network, on different dates, through social media (WhatsApp, Instagram, and Facebook), to representatives of Palliative Care services working in Brazil that attended the pediatric age group. The online recruitment was made using links to the questionnaire, in a Google Form, and this ran for four months (between February 2021 and May 2021). Only one representative per service was accepted. Services being defined as hospitals, clinics, outpatient clinics, health units and home care groups, the inclusion criteria were being a palliative care service that attended to pediatrics, that is, children from zero to 18 years of age. The exclusion criteria were new entries from the same service.

### 2.3 The questionnaire

The questionnaire mostly included quantitative questions. Some open questions were also asked, to complement analyses. It was produced by the researchers, based on experience and complement information already found in other studies and documents (6, 10–12) and was composed of four components: identification and characterization of the services themselves such as state, service opening date, level of care, number of beds, among others), characterization of the health professionals who work in PPC in these services (formation of the team, whether the coordinator has specialization, time each professional dedicates to PPC, among others), access to opioid prescriptions in these locations (if opioids are easy to prescribe, if there is any difficulty in accessing them, for example) and education in PPC offered (If there is a linked pediatric residency, if there is continuing education for professionals, for example), both for new professionals and residents, as well as continuing education for health professionals already working there.

The questionnaire’s functionality was previously tested among all the researchers. The questionnaires were anonymous. Only the institution, the service name, and the e-mail address were registered. All IP addresses were checked for duplicates. Only the coordinator of the survey had access to all questionnaire answers. All data was stored in a database on a password-protected computer.

### 2.4 Statistical analysis

Descriptive qualitative analysis was performed, and frequencies and 95% confidence intervals (95% CI) were calculated. The results are presented as graphs and tables. Comparisons were made between the services that attended oncologic patients and the others were made. Differences between proportions are determined by the Fisher exact or chi-squared test. All analyses were performed using Stata version 18.0 (Stata Corp, *L.C.*) We built a word cloud about the commentaries made by the services attending oncologic patients with a word cloud generator (google.com).

### 2.5 Ethic

This study followed the CROSS guideline (13) for reporting survey studies and was approved by the Research Ethics Committee (CAAE 39915620.2.0000.5504). All participants signed the Informed Consent Form, before answering the survey. This study was performed in line with the principles of the Declaration of Helsinki.

## 3 Results

All services were made up of at least two health professionals with different training, that is, interdisciplinary teams. Of the 90 services that answered the questionnaire, 40 (44.4%) attended pediatric oncology patients. Ten services were created before 2009, and the remaining services were created after 2010. São Paulo represented the largest state, with 42.22% (38 services), followed by Minas Gerais with 8.89% (8 services). The Southeast represented most of the services (57.57%), followed by the Northeast, with 18.89% (17 services), the South with 12.22% (11 services), and the Center West with 8.89% (8 services). We observed that services with oncology care had a higher frequency of pediatric intensive care beds than services that cared for non-oncology patients. More details can be seen in **Table 1**.

**Table 1:** Characteristics of services that attended oncologic patients and the others.

Characteristics of services			
	Services with oncologic patients (n=40)	Services without oncologic patients (n=50)	p-value*
**State where the service is located, n (%)** São Paulo Paraná Minas Gerais Ceará Rio de Janeiro Distrito Federal Outros	17 (42.5) 4 (10.0) 3 (7.5) 3 (7.5) 1 (2.5) 1 (2.5) 11 (27.5)	21 (42.0) 2 (4.0) 5 (10.0) 4 (8.0) 3 (6.0) 4 (8.0) 11 (22.0)	0.32
**Administration, n (%)** Only Public Only Private Only Philanthropic Other	20 (50.0) 8 (20.0) 5 (12.5) 7 (17.5)	36 (72.0) 9 (18.0) 3 (6.0) 2 (4.0)	0.15
**Level of care, n (%)** Primary Secondary Tertiary Quaternary	1 (2.5) 8 (20.0) 3 (7.5) 28 (70.0)	2 (4.0) 8 (16.0) 11 (22.0) 29 (58.0)	0.27
**Linked to a university, n (%)** Yes, public Yes, private No	13 (32.5) 5 (12.5) 22 (55.0)	15 (30.0) 9 (18.0) 26 (52.0)	0.77
**Number of pediatric and neonatal beds, n (%)** <50 Between 50 and 100 Between 100 and 200 >200 None	17 (42.5) 12 (30.0) 4 (10.0) 4 (10.0) 3 (7.5)	17 (34.0) 17 (34.0) 5 (10.0) 5 (10.0) 6 (12.0)	0.91
**Exclusive beds for Pediatric Palliative Care, n (%)** Yes No	6 (15.0) 34 (85.0)	6 (12.0) 44 (88.0)	0.67
**Pediatric Intensive Care beds, n (%)** Yes No	34 (85.0) 6 (15.0)	33 (66.0) 17 (34.0)	**0.04**

*p-value associated with chi-squared test.

Reinforces that these values were significant.

We compared healthcare team characteristics between the services that attended oncologic patients and the others and observed a tendency for those services that attended oncologic patients to dedicate more time to Pediatric Palliative Care. It is interesting to note that the majority of services in Brazil are public, reinforcing the importance of the single health service in the country. There are more public services in non-cancer care. In PPC, there is a great demand for primary and secondary services in non-oncology care, but the majority of services are tertiary and quaternary, which demonstrates a specificity even in services of greater complexity even in non-oncology care: more features are seen in **Table 2**.

**Table 2:** Healthcare team characteristics according to services (n=90).

Healthcare team			
	Services with oncologic patients (n=40)	Services without oncologic patients (n=50)	p-value*
**The team is exclusive for Pediatric Palliative Care, n (%)** Yes No	11 (27.5) 29 (72.5)	11 (22.0) 39 (78.0)	0.54
**The Pediatric Palliative Care team is exclusive for Pediatrics, n (%)** Yes No	22 (55.0) 18 (45.0)	35 (70.0) 15 (30.0)	0.14
**Mean weekly time dedicated to Pediatric Palliative Care, n (%)** <10 hours Between 10 and 30 hours >30 hours	16 (40.0) 20 (50.0) 4 (10.0)	30 (60.0) 19 (38.0) 1 (2.0)	0.08

*p-value associated with chi-squared test.

Comparing the existence of grief support to patients, we observed no differences between the services (67.5% *versus* 60.0%, p-value=0.46). No differences were observed in access to opioid prescriptions between the services. In the services without oncologic attendance, 26 services had total access to opioid prescription, 21 had some access and none had no access, versus 28, 11 and 1 services, respectively, with oncologic attendance (p-value=0.20).

Regarding educational and research questions, 55% of oncology services have pediatric residency programs, while 58% of other services also have these programs. Initiatives aimed at training health professionals, that is, continuing education for the team, were found in 62.5% of oncology services and 78% in the others. Regarding PPC research, 27% of oncology services carry out research activities, compared to 36% in non-oncology services. These results with p-value associated with the chi-squared test can be seen in **Table 3**.

**Table 3:** Healthcare professionals’ education and development of research on Pediatric Palliative Care.

Education and Research			
	Services with oncologic patients (n=40)	Services without oncologic patients (n=50)	p-value*
**Pediatrics Residency Program, n (%)** Yes No	22 (55.0) 18 (45.0)	29 (58.0) 21 (42.0)	0.77
**Initiatives turned to healthcare professionals’ education, n (%)** Yes No	25 (62.5) 15 (37.5)	39 (78.0) 11 (22.0)	0.10
**Research on Pediatric Palliative Care, n (%)** Yes No	11 (27.5) 29 (72.5)	18 (36.0) 32 (64.0)	0.39

*p-value associated with chi-squared test.

## 4 Discussion

### 4.1 Reflections

In general, ten services were created before 2009, compared to 80 services that were created after. In other words, Pediatric Palliative Care is recent in Brazil. The distribution of services that cover oncology and those that do not, are similar in the different regions of Brazil, the location of services was found expressively in areas with more economic development: regional disparities within a country of continental dimensions are reflected by these differences. It was found that there were more pediatric intensive care beds in services that attended oncologic patients. Access to opioid prescriptions without differences revealed that even pediatric oncologists might have difficulty with this prescription, and no differences were observed between the services, regarding education and research.

### 4.2 Gaps in distribution

The distribution of services that cover oncology and those that do not, are similar in the different regions of Brazil. A recently published article demonstrated that there are very important regional differences in the number of PPC services in the country, which does not mean that there are no children in need of this attention in these locations (4).

In this study, the location of services was found expressively in areas with more economic development, in a country with continental dimensions and disparities, suggesting that the areas not included might have gaps in treatment in PPC and oncology (14).

### 4.3 Specificities of oncology services

In this research, it was found that there were more pediatric intensive care beds in services that attended oncologic patients, which probably shows more resources in these locations. A recent study demonstrated that places with PPC teams had a higher number of beds in general, with the median number being 185 beds versus 49 beds for those places without PPC teams (15). It was also seen that facilities with a higher proportion of trauma, intensive care or acuity levels were more likely to offer PPC (15).

It was also seen as a tendency to dedicate more time to PPC in services that attend oncologic patients. Integrating palliative care into routine care for children and adolescents with cancer has resulted in better outcomes for patients and their families (2). The field of pediatric palliative oncology, encompassing primary palliative care provided by the multidisciplinary oncology team as well as subspecialty palliative care provided by the palliative care team for more complex cases, is unique from adult palliative care because of its focus on the care of the child and the family (2). This article demonstrates that the key point in Brazil is the urgent need for a definition of a palliative team in services that attend to oncologic patients.

### 4.4 Pediatric pain care and opioids

There is strict control by health authorities in Brazil for prescribing opioids (16): to prescribe opioids in Brazil, a special yellow prescription is required, in which the medical professional or the service’s technical manager needs to carry out specific bureaucracy, with each city having its own specific rule. As this process is not simple and requires teaching in the area, it is not uncommon for there to be difficulties in prescribing opioids in the country.

Understanding that oncology services are the ones that prescribe the most opioids, but that prescriptions for non-cancer pain have been increasing in Brazil, the hypothesis would be that there would be fewer difficulties in PPC services that serve oncology compared to those that do not treat oncology (16). Regarding access to opioids in pediatrics, Brazil is below what is necessary. While Palliative Care services in general present 7.9% difficulties in accessing opioids, PPC services present 40% difficulties or no access (4). At the same time, pediatric oncology teams can be considered services that frequently deal with complex pain in children (17), therefore, they should have fewer difficulties accessing opioids: the access to opioid prescriptions without differences found in this study revealed that even pediatric oncologists might have difficulty with this prescription, and this should improve. Symptom control at the end of life, especially in pediatrics, is a difficult and complex management process that requires training in PPC and not just in oncology (18).

### 4.5 Education in pediatric pain and PPC

In this study, no differences were observed between the services, regarding education and research. Education is the key to improving PPC and to developing services and capacities, especially in countries with limited resources (19), a place where Brazil still is (20). The emphasis should be given to palliative care (20), and PPC (1) and related areas (21) in academic and professional training, and further studies in search of the best evidence should be conducted to support Evidence-Based Practices (1, 19).

### 4.6 Limitations

This research has limitations. Specifics about the services were not researched at this time, concluding with answers still pending, such as the number of consultations per period or the format in which the teams operate. Also, some services may not have been found. Healthcare in Brazil can be difficult to contextualize, as it is a country with continental dimensions and important social disparities, including between different regions, which can impair the understanding of the text. Our research did not focus on evaluating the quality of services, but rather on some aspects: this is a limitation of this study. Research funding agencies in our country do not see PPC issues as important, which is an immense limitation for us to take quality research in our country to the world. There are services that do not have essential professionals, such as chaplains and physical education professionals: this is a great difficulty in Brazil, including defining a minimum team. In Brazil, training is primarily carried out by medical residencies, which makes it difficult to understand whether there are other types of training.

## 5 Conclusions

Brazil is a country of continental dimensions but, along with this, carries significant disparities. Therefore, children with oncological diseases are possibly not being included in the PPC. Even with the tendency to dedicate more time to PPC in services that serve oncology patients, access to opioid prescriptions with no differences compared to services that do not treat oncology revealed that, even pediatric oncologists, may have difficulties with this prescription. In fact, the need to define a palliative team is urgent in services that care for cancer patients. In this study, no differences were observed between services with regard to teaching and research; therefore, it is important to emphasize that education is the key to improving PPC and developing capabilities, especially in areas with limited resources.

## Data availability statement

The original contributions presented in the study are included in the article/Supplementary Material. Further inquiries can be directed to the corresponding author.

## Ethics statement

The studies involving humans were approved by Research Ethics Committee of the Federal University of São Carlos (CAAE number 39915620.2.0000.5504). The studies were conducted in accordance with the local legislation and institutional requirements. The participants provided their written informed consent to participate in this study.

## Author contributions

EF: Conceptualization, Data curation, Formal analysis, Funding acquisition, Investigation, Methodology, Project administration, Resources, Software, Supervision, Validation, Visualization, Writing – original draft, Writing – review & editing. CV: Data curation, Formal analysis, Funding acquisition, Investigation, Methodology, Project administration, Resources, Software, Validation, Visualization, Writing – original draft, Writing – review & editing. SB: Data curation, Funding acquisition, Investigation, Methodology, Project administration, Resources, Validation, Visualization, Writing – original draft, Writing – review & editing. GC: Data curation, Funding acquisition, Investigation, Methodology, Project administration, Resources, Validation, Visualization, Writing – original draft, Writing – review & editing. PM: Data curation, Funding acquisition, Investigation, Methodology, Project administration, Resources, Validation, Visualization, Writing – original draft, Writing – review & editing. AC: Data curation, Funding acquisition, Investigation, Methodology, Project administration, Resources, Validation, Visualization, Writing – original draft, Writing – review & editing. SI: Data curation, Funding acquisition, Investigation, Methodology, Project administration, Resources, Validation, Visualization, Writing – original draft, Writing – review & editing. MS: Data curation, Funding acquisition, Methodology, Resources, Validation, Visualization, Writing – original draft, Writing – review & editing. RR: Funding acquisition, Methodology, Resources, Validation, Visualization, Writing – original draft, Writing – review & editing. CB: Funding acquisition, Methodology, Resources, Validation, Visualization, Writing – original draft, Writing – review & editing. MG-C: Funding acquisition, Resources, Validation, Visualization, Writing – original draft, Writing – review & editing. AU: Funding acquisition, Resources, Validation, Visualization, Writing – original draft, Writing – review & editing.
